# Development of a real-time loop-mediated isothermal amplification method for monitoring *Pseudomonas lurida* in raw milk throughout the year of pasture

**DOI:** 10.3389/fmicb.2023.1133077

**Published:** 2023-04-12

**Authors:** Shufei Zhang, Lianxia Hu, Yuling Xue, Dong Zhang, Yaoguang Zhang, Shijie Wang

**Affiliations:** ^1^College of Food Science and Biology, Hebei University of Science and Technology, Shijiazhuang, Hebei, China; ^2^College of Chemical Engineering, Shijiazhuang University, Shijiazhuang, Hebei, China; ^3^Junlebao Dairy Group Co., Ltd., Shijiazhuang, Hebei, China

**Keywords:** gyrB gene, aprX gene, real-time LAMP, *Pseudomonas lurida*, thermostable alkaline proteases, raw milk

## Abstract

**Introduction:**

The psychrophilic bacterium *Pseudomonas lurida* (*P. lurida*) and its thermostable alkaline proteases can seriously damage raw milk quality.

**Methods:**

In this study, specific primers were designed for *P. lurida’s gyrB* and *aprX* genes, and a real-time loop-mediated isothermal amplification (RealAmp) rapid detection method was developed for the early monitoring of *P. lurida* and its proteases in raw milk. A phylogenetic tree of the *gyrB* and *aprX* genes of *P. lurida* was constructed to analyze the homology of the design sequence of the RealAmp primer. The DNA of 2 strains of *P. lurida* and 44 strains of non-*P. lurida* were detected *via* RealAmp to analyze the specificity of the primer.

**Results:**

It was found that *aprX*-positive proteases were produced by *P. lurida-*positive strains only when *Pseudomonas fluorescens* was negative. The dissociation temperatures of *gyrB* and *aprX* in the RealAmp-amplified products were approximately 85.0°C and 90.0°C, respectively. Moreover, DNA was detected through a 10-fold dilution of *P. lurida* in a pure bacterial solution and artificially contaminated skimmed milk. The limit of detection of *P. lurida DNA* copy number in the pure bacterial solution was 8.6 copies/μL and that in the 10% skimmed milk was 5.5 copies/μL. Further, 144 raw milk samples throughout the year from three farms in Hebei province were analyzed using RealAmp. The highest detection rate of *P. lurida* was 56% in the first and third quarters, and that of proteases was 36% in the second quarter. The detection rates of *P. lurida* and its proteases were the highest in samples collected from pasture 2 (52 and 46%, respectively), and the ability of *P. lurida* to produce proteases reached 88%.

**Discussion:**

In conclusion, RealAmp established an early and rapid method for the detection of *P. lurida* and its proteases in raw milk samples, allowing the identification and control of contamination sources in a timely manner to ensure the quality of milk and dairy products.

## 1. Introduction

*Pseudomonas lurida* (*P. lurida*), a new fluorescent species of the *Pseudomonas* genus associated with the phyllosphere of grasses, was first isolated by Behrendt et al. in (2007). *Pseudomonas lurida* is a non-spore-forming, fluorescent, Gram-negative, and plant pathogenic bacterium (Mishra et al., 2009; Selvakumar et al., 2011). It reportedly exhibits pathogenic and damaging characteristics in citrus leaves and stems (Beiki et al., 2016; Yuan et al., 2018; Guo, 2020; Du, 2022). Further, it is considered a psychrophilic bacterium (Bisht et al., 2015; Meng et al., 2018) that produces thermostable alkaline proteases and lipases that spoil milk and dairy products (Khannous et al., 2014; Quintieri et al., 2020). For example, *Pseudomonas fluorescens* are thermostable alkaline proteases produced by *P. lurida* which are the primary determinant of quality in milk and dairy products during storage (Quintieri et al., 2020); even after ultra-high temperature instantaneous sterilization at 137°C for 4 s, 10% of the alkaline proteases remain active (Baglinière et al., 2013). Therefore, the monitoring and control of *P. lurida*-produced thermostable alkaline proteases are essential to ensure high quality of raw milk and dairy products.

The *Pseudomonas* genus is a large group divided into five categories (Bossis et al., 2000) with target genes, including the *apr* gene (Dufour et al., 2008), *gyrB* gene (Bablishvili et al., 2017), and intergenic spacer sequence of 16S-23S rRNA (Hu et al., 2022). The *gyrB* gene (Lu et al., 2016) is a protein-coding gene that is related to DNA replication, modification, restriction, and repair. It can be used to distinguish *Pseudomonas* species and even members of the same species by using the restrictive fragment length polymorphism method (Agaras and Valverde, 2018). The *apr* gene (D’Incecco et al., 2019) is a thermostable-alkaline-protease-coding gene, which exists in *Pseudomonas* and other spoilage bacteria that can hydrolyze proteins. Notably, a previous study engineered *aprX* gene primers based on *Pseudomonas* and detected the thermostable alkaline proteases of *P. fluorescens* (Hu et al., 2022). *P. lurida* is usually identified using a combination of traditional isolation and culture methods (3–4 d) and 16S rRNA sequencing (1 d, excluding transportation time; Behrendt et al., 2007). However, *P. lurida* has a long culture cycle, making it difficult to achieve accurate risk assessment. Loop-mediated isothermal amplification (LAMP) is a method for rapid amplification of nucleic acids at a constant temperature (Notomi et al., 2000), where two pairs of primers are designed to identify and bind to six different regions on the template gene sequence. The amplification reaction is performed using the primers and a DNA polymerase with high chain activity and can produce up to 10^9^ copies of the amplified DNA within 1 h (Yang et al., 2018). This method has several advantages, such as strong specificity, high sensitivity, simple operation, rapid reaction, and low cost; therefore, it is widely used in various fields (Iwamoto et al., 2003). In addition, real-time fluorescence LAMP (RealAmp) technology has been established by adding SYBR Green I dye to the system before the reaction, which allows the data to be analyzed based on the amplification curve and peak time (Hu et al., 2022). However, no RealAmp reaction system has been developed for *P. lurida* and its thermostable alkaline proteases detection to monitor their dynamic changes in raw milk throughout the year of pasture.

The present study aimed to develop a RealAmp reaction system for the early and rapid detection of *P. lurida* and its thermostable alkaline proteases in raw milk. Primers were designed based on the *apr*X (Hu et al., 2022) and *gyrB* genes of *P. lurida*. and the RealAmp reaction system and conditions were optimized. This method allowed the timely detection of *P. lurida* and its proteases to ensure the quality of raw milk and dairy products.

## 2. Materials and methods

### 2.1. Strain culture

Two strains of *P. lurida* and 44 related strains were detected using RealAmp to analyze the specificity of the primers: *P. lurida* (CICC22026 and CICC22027), *Pseudomonas putida* (CICC21624, ATCC12633, ATCC23483, and ATCC17485), *Pseudomonas aeruginosa* (CICC10351, ATCC15692, and ATCC27853), *P. fluorescens* (CICC23250, CICC23251, CICC23254, and ATCC13525), *Pseudomonas stutzeri* (CICC10402 and CICC23002), *Pseudomonas rhodesiae* (CICC21957, CICC22694 and CICC22695), *Pseudomonas simiae* (CICC22692), *Pseudomonas alcaligenes* (CICC23927, CICC23069 and CICC25146), *Pseudomonas chlororaphis* (CICC10216, CICC21627, and ATCC13986), *Bacillus licheniformis* (CICC10037, CICC22068, ATCC14580, and ATCC21424), *Bacillus cereus* (CICC20450, CICC10468, ATCC11778, and ATCC14579), *Staphylococcus aureus* (CICC10201, CICC10145, ATCC6538 and ATCC25923), *Listeria monocytogenes* (ATCC19111, ATCC19115, and ATCC51772), *Cronobacter sakazakii* (CICC24112, CICC24125, and ATCC29544), and *Salmonella typhimurium* (CICC21483, CICC21484, and CICC21913). *Pseudomonas lurida* and *Pseudomonas* strains were cultured in a medium of Brain Heart Infusion broth for 18 h at 30°C. The pure cultures of nine non-*Pseudomonas* strains were incubated in nutrient broth at their optimum temperatures for 18 h.

### 2.2. DNA extraction method

Deoxyribonucleic acid (DNA) was extracted using a bacterial DNA extraction kit and boiling water bath. The DNA extraction kit purchased from TIANGEN Biotech C., Ltd. (Beijing, China) and was used according to the manufacturer’s protocol. The boiling water bath method was previously used in real-time LAMP identification of *P. fluorescens*-produced thermostable alkaline proteases (Hu et al., 2022).

### 2.3. Design and synthesis of RealAmp primers

RealAmp primers for the *gyrB* gene of *P. lurida* (GenBank: MT941328.1) were designed using Primerexplorer V5, an online primer-designing software.

The outer primer of forward (Q-F3) was 5′-TGGGAACAGACCTACGT-3′ and backward (Q-B3) was 5′-GGAAGGACAGTTCACG-3′. The inner primer of forward (Q-FIP) was 5′-GCGTACCGGTGCCACGGTGTTCCACAGGA-3′ and backward (Q-BIP) was 5′-CATTTCAAGCCATCGGCTGATACGCTTGGCCAGGATGT-3′. The loop primer of forward (Q-LF) was 5′-GTCGCCGACGATTTTCATC-3′ and backward (Q-LB) was 5′-CCTTCAAGAATATCCACT-3′.

The primer sequences of the *aprX* gene of *P. lurida* were obtained from the study of Hu et al. (2022). The outer primer of forward (L-F3) was 5′-GCCCGCTGATCGACGACAT-3′ and backward (L-B3) was 5′-AGTCCAGGGTGTCGTTGCC-3′. The inner primer of forward (L-FIP) was 5′-AGGTGGTGTCCGTGGCGCGATCCAGAAGCTCTA-3′ and backward (L-BIP) was 5′-GGTTCAACTCCAACACCGCCGTCCCATACCGAGAACA-3′. The loop primer of forward (L-LF) was 5′-GCTGAGGTTGGCACCG-3′ and backward (L-LB) was 5′-GCTACTTCCAATGCCGACA-3′. The primers were synthesized by General Biosystems (Anhui, China).

### 2.4. Reaction system and conditions

The *P. lurida* RealAmp reaction system based on the *gyrB* gene was as follows: 2 mM of MgSO_4_, 0.4 mM of dNTP, 1.2 μM each of Q-FIP and Q-BIP, 0.2 μM each of Q-F3 and Q-B3, 0.4 μM each of Q-LF and Q-LB, 0.2 M of betaine (Sigma-Aldrich, MO, USA), 2.5 μL of 10× ThermoPol Buffer, 8 U of Bst polymerase (New England Biolabs Inc., Ipswich, Massachusetts, USA), 1/300 dilution of 0.3 μL of 10,000× SYBR green I, 1 μL of DNA template, and added sterilized distilled water up to a final volume of 25 μL.

The *P. lurida* RealAmp reaction system based on the *aprX* gene was as follows: 2 mM of MgSO_4_, 0.4 mM of dNTP, 1.4 μM each of L-FIP and L-BIP, 0.28 μM each of L-F3 and L-B3, 0.28 μM each of L-LF and L-LB, 0.2 M of betaine (Sigma-Aldrich), 2.5 μL of 10× ThermoPol Buffer, 8 U of Bst polymerase (New England Biolabs Inc.), 1/300 of dilution of 0.3 μL of 10,000× SYBR green I, 1 μL of DNA template, and added sterilized distilled water up to a final volume of 25 μL.

Further, 20 μL of mineral oil was used to cover the reaction system to prevent contamination. For the *gyrB* and *aprX* genes, reaction tubes were held at 61°C and 62°C, respectively, for 40 min in the fluorescent quantitative PCR instrument (QuantStudio 3, Applied Biosystems, Waltham, MA, USA). After the RealAmp reaction was complete, the detection result was determined based on the peak time.

### 2.5. Construction of phylogenetic trees of the *gyrB* and *aprX* genes of *Pseudomonas lurida*

Two phylogenetic trees were constructed separately targeting the amplified regions of the *gyrB* and *aprX* genes of *P. lurida* and the sequences with more than 95 and 94% identity with non-*P. lurida* strains in the GenBank, respectively. By separately aligning the *gyrB* and *aprX* sequences of the genus *Pseudomonas* and their related species from the GenBank using the Clustalx1.81 software, 38 sequences of each were determined. After these sequences were aligned using the DAMBE software, the similar sequences were removed. Finally, 19 unique sequences of *gyrB* and 30 unique sequences of *aprX* were retained, which were used to construct their respective phylogenetic trees.

### 2.6. Assessment of assay specificity

To determine the specificity of the RealAmp assay for *gyrB* and *aprX* genes of *P. lurida,* the DNA of 2 *P. lurida* and 44 related strains was extracted and used as the template for RealAmp amplification. The specificity of the RealAmp primers of *P. lurida gyrB* and *aprX* genes was verified by observing the amplification curve and the corresponding peak of dissociation temperatures. The blank control DNA was replaced with sterilized distilled water, and all experiments were repeated five times.

### 2.7. Limit of detection of *Pseudomonas lurida* in pure cultures

The bacterial suspension of *P. lurida* was quantified, which was found to be 5.6 × 10^8^ colony-forming units (CFU)/mL. DNA was extracted from 1 ml of Brain Heart Infusion broth with *P. lurida* cultured after 18 h at 30°C. The concentration of the extracted DNA was approximately 5.75 fg/μL–57.5 ng/μL after a 10× gradient dilution. The content of *P. lurida* was 5.6 × 10^1^–5.6 × 10^8^ CFU/ml. The RealAmp reaction was performed using *P. lurida* DNA copy number with a 10× gradient variation of approximately 8.6 × 10^0^–8.6 × 10^6^ copies/μL as the template, and the detection limit of *P. lurida* in pure bacterial solution *via* RealAmp method was verified according to the peak time. The formula for calculating copies/μL was as follows:


$$copies/\mu L=\frac{\left(6.02\times 10^{23}\right)\;copies/moL\times \left(DNA\;concentration\right)\;ng/\mu L\times 10^{-9}\;}{\left(DNA\;lengthof\;P.lurida\right)\;bp\times \left(660\;\mathrm{ng}/\mathrm{moL}\right)\;bp^{-1}}$$

DNA length of *P. lurida in* the formula was approximately 6.1 × 10^6^ bp. Under the same conditions, 10 measurements were performed, and their mean and standard deviation (SD) were calculated. The repeatability of the method was evaluated by obtaining the variation coefficient (CV) of the peaking time (Ct) according to the following formula:


$$CV=\frac{SD}{mean}\times 100\%$$

### 2.8. Limit of detection for artificially added *Pseudomonas lurida* in skimmed milk

Sterile skimmed milk (10%) was inoculated on KB plates and cultured at 30°C for 48 h. As no subsequent colony growth was observed, the skimmed milk was confirmed to be free of *P. lurida* contamination before adding *P. lurida*. The bacterial suspension of *P. lurida* (4 ml) was centrifuged at 13,523× *g* for 2 min. The collected bacteria were resuspended in sterile water (1 ml) as the original bacterial solution and added to 10% sterile skimmed milk (9 ml), after which plate counting was performed. The content of *P. lurida* in artificially contaminated 10% skimmed milk was 2.4 × 10^8^ CFU/ml. Further, 1 ml of the artificially contaminated 10% skimmed milk was used to extract the bacterial DNA. After 10× gradient dilution of the extracted DNA, the DNA concentration was approximately found to be 3.70 fg/μL–37.0 ng/μL. The corresponding *P. lurida* content was 2.4 × 10^1^–2.4 × 10^8^ CFU/ml. The RealAmp reaction was performed using different DNA copy number with a 10× gradient variation of approximately 5.5 × 10^0^–5.5 × 10^6^ copies/μL as the template to determine the limit of detection for *P. lurida* in artificially contaminated milk samples. Under the same conditions, 10 measurements were performed, and their mean and SD were calculated. The CV of the Ct was calculated using the above-mentioned formula.

### 2.9. Detection of *Pseudomonas lurida* in raw milk samples

Using sterile centrifuge tubes, a total of 144 raw milk samples were collected for a year from the cows in three different pastures in Hebei, China. The first pasture (east longitude 114°31′10.88″ and north latitude 37°45′57.65″) contained 463 cows; the second (east longitude 114°39′57.93″ and north latitude 38°15′57.82″) contained 388 cows; and the third (east longitude 115°43′11.27″ and north latitude 38°14′6.90″) contained 342 cows. Raw milk was collected once a week from the mixed milk samples (about 4–6 tons per pasture) obtained from all cows in each pasture in 1 day. All cows in each pasture were milked three times a day: in the morning, middle of the day, and evening, and the fresh milk from each collection was temporarily stored in a storage tank at 4°C. All samples collected in 1 day were combined into a mixed milk sample, which was then sent to the milk and dairy processing plant. Each sample was divided into three parts, two of which were used for the separate detection of both genes, and one was retained at −80°C for storage. Each raw milk sample (1 ml) was collected into a centrifuge tube (2.0 ml), to which anhydrous ethanol (0.2 ml), ammonia (0.2 ml), and petroleum ether (0.2 ml) were added, mixed, and centrifuged at 13,523× *g* for 10 min. The upper fat and supernatant were discarded, 50 μL Tris-EDTA buffer solution (TE) was added to the precipitate, and the mixture was shaken and then boiled at 100°C for 10 min. Subsequently, it was resuspended and centrifuged at 13,523× *g* for 5 min, following which the supernatant (DNA) was collected and stored at −20°C. RealAmp detection was performed using the DNA extracted from 144 raw milk samples. The primers designed for the *apr*X gene were used to detect proteases (Hu et al., 2022).

### 2.10. Statistical analysis

SPSS 26.0 was used for statistical analysis. The Kruskal–Wallis test for nonparametric data was used for the statistical analysis of data that did not conform to normal distribution.

## 3. Results

### 3.1. Phylogenetic trees of *Pseudomonas lurida* and related species targeting the *gyrB* and *aprX* genes

**Figure 1:**
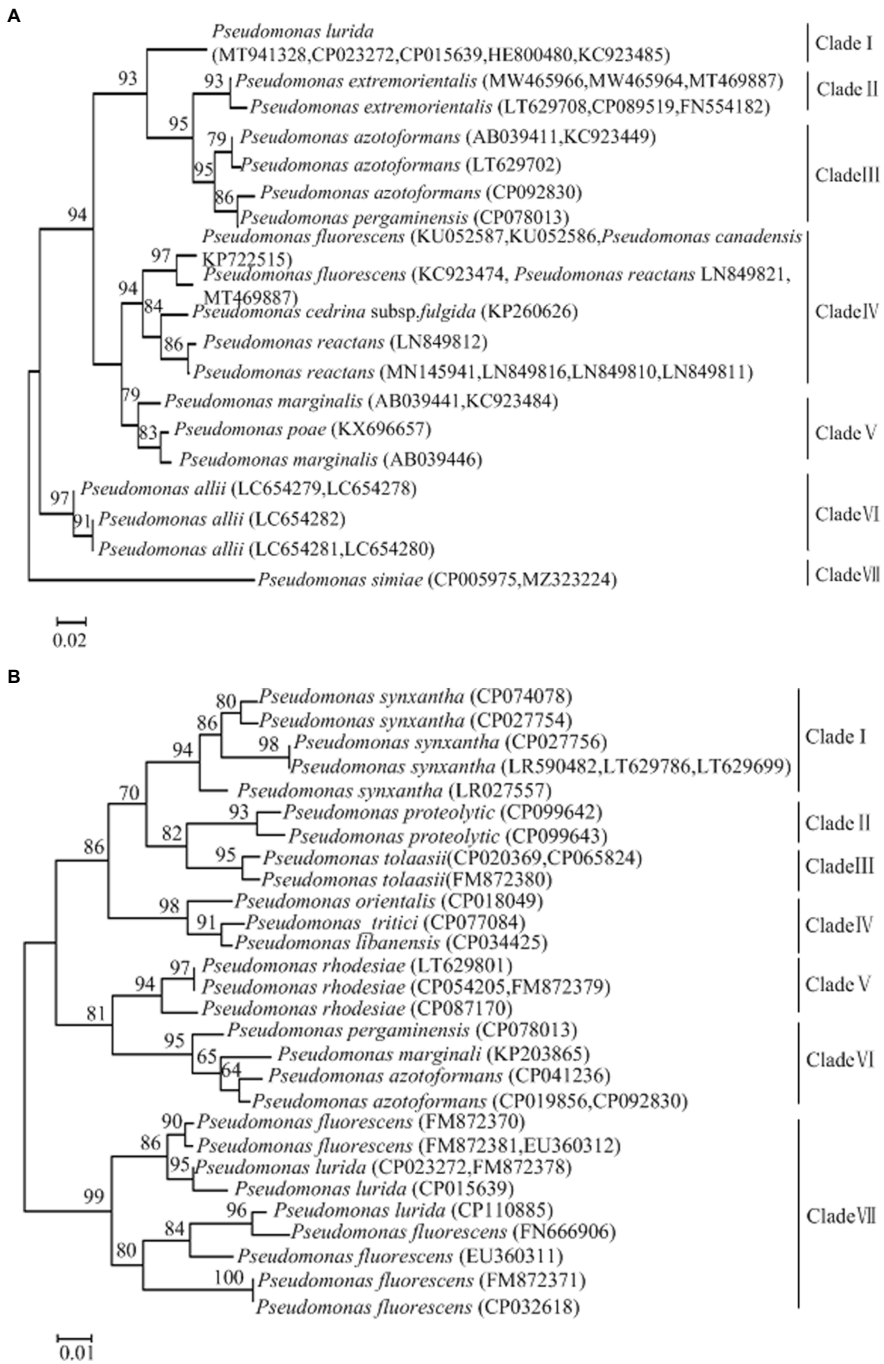
**(A)** Phylogenetic trees of *Pseudomonas lurida* and related species targeting the *gyrB* genes. **(B)** Phylogenetic trees of *Pseudomonas lurida* and related species targeting the *aprX* genes.

A phylogenetic tree of the genus *Pseudomonas* targeting the *gyrB* gene, was constructed using the neighbor-joining method (Figure 1A). This tree was divided into seven main clades, Clade I–VII. Clade I contained *P. lurida*; Clade II contained *Pseudomonas extremorientalis*; Clade III contained *Pseudomonas azotoformans* and *Pseudomonas pergaminensis*; Clade IV contained *P. fluorescens*, *Pseudomonas cedrina* subsp*. fulgida*, and *Pseudomonas reactans*; Clade V contained *Pseudomonas marginalis* and *Pseudomonas poae*; Clade VI contained *Pseudomonas allii*; and Clade VII contained *P. simiae*. Further, five strains of *P. lurida* clustered on the same branch. The phylogenetic tree showed that the initial *gyrB* gene sequence (166 bp) of *P. lurida* amplified by RealAmp had high intraspecific homology with *P. lurida* species and low interspecific homology with other *Pseudomonas* species.

A phylogenetic tree of the genus *Pseudomonas* targeting the *aprX* gene, was constructed using the neighbor-joining method (Figure 1B). This tree was divided into seven main clades, Clade I–VII. Clade I contained *Pseudomonas synxantha*; Clade II contained *Pseudomonas proteolytica*; Clade III contained *Pseudomonas tolaasii*; Clade IV contained *Pseudomonas orientalis*, *Pseudomonas tritici*, and *Pseudomonas libanensis*; Clade Vcontained *P. rhodesiae*; Clade VI contained *P. pergaminensis*, *P. marginalis*, and *P. azotoformans*; and Clade VII contained *P. lurida* and *P. fluorescens.* Further, *P. lurida* and *P. fluorescens* were clustered in the same branch, indicating that the initial *aprX* gene sequence (183 bp) of *P. lurida* amplified by RealAmp had high homology with *P. lurida* and *P. fluorescens*. The phylogenetic tree showed that *P. lurida* and *P. fluorescens* could not be distinguished between themselves, but they could be distinguished from other *Pseudomonas* species.

### 3.2. Specificity of RealAmp assay

**Figure 2:**
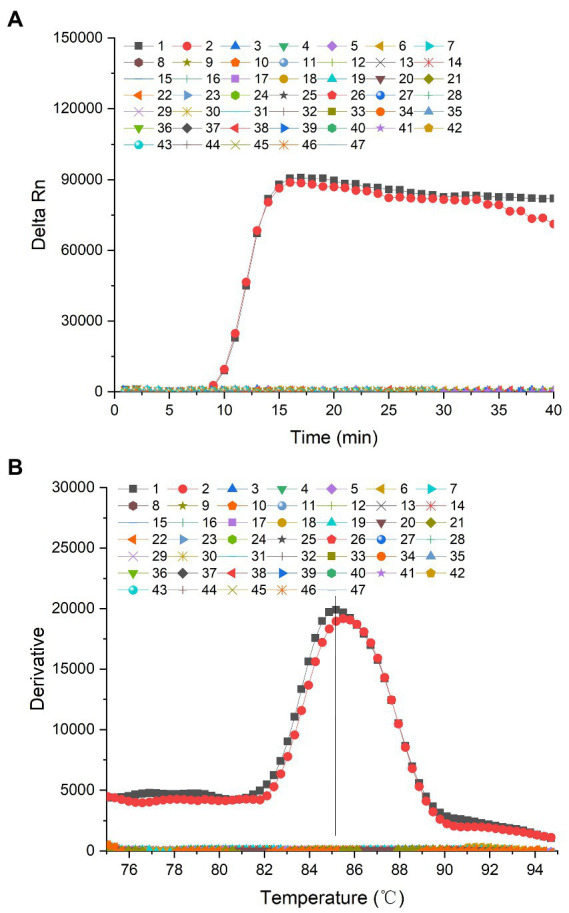
**(A)** Amplification curve of the specificity of the *gyrB* gene of *Pseudomonas lurida*. 1: *P. lurida* CICC22026, 2: *P. lurida* CICC22027, 3: *Pseudomonas fluorescens* ATCC13525, 4: *P. fluorescens* CICC23250, 5: *P. fluorescens* CICC23251, 6: *P. fluorescens* CICC23254, 7: *Pseudomonas aeruginosa* CICC10351, 8: *P. aeruginosa* ATCC 27853, 9: *P. aeruginosa* ATCC 15692, 10: *Pseudomonas alcaligenes* CICC23927, 11: *P. alcaligenes* CICC 23069, 12: *P. alcaligenes* CICC25146, 13: *P. simiae* CICC22692, 14: *Pseudomonas stutzeri* CICC10402, 15: *P. stutzeri* CICC23002, 16: *Pseudomonas chlororaphis* CICC21627, 17: *P. chlororaphis* ATCC 13986, 18: *P. chlororaphis* CICC 10216, 19: *Pseudomonas rhodesiae* CICC21957, 20: *P. rhodesiae* CICC 22694, 21: *P. rhodesiae* CICC 22695, 22: *P. putida* ATCC17485, 23: *P. putida* CICC21624, 24: *P. putida* ATCC 12633, 25: *P. putida* ATCC 23483, 26: *Bacillus cereus* CICC20450, 27: *B. cereus* CICC10468, 28: *B. cereus* ATCC 14579, 29: *B. cereus* ATCC 11778, 30: *Bacillus licheniformis* ATCC21424, 31: *B. licheniformis* CICC10037, 32: *B. licheniformis* CICC22068, 33: *B. licheniformis* ATCC 14580, 34: *Staphylococcus aureus* ATCC25923, 35: *S. aureus* ATCC6538, 36: *S. aureus* CICC 10145, 37: *S. aureus* CICC 10201, 38: *Listeria monocytogenes* ATCC19111, 39: *L. monocytogenes* ATCC 51772, 40: *L. monocytogenes* ATCC 19115, 41: *Cronobacter sakazakii* ATCC29544, 42: *C. sakazakii* CICC 24112, 43: *C. sakazakii* CICC 24125, 44: *Salmonella typhimurium* CICC21484, 45: *S. typhimurium* CICC 21913, 46: *S. typhimurium* CICC 21483, 47: Blank control. **(B)** Dissociation curve of the specificity of the *gyrB* gene of *Pseudomonas lurida*. 1: *P. lurida* CICC22026, 2: *P. lurida* CICC22027, 3: *Pseudomonas fluorescens* ATCC13525, 4: *P. fluorescens* CICC23250, 5: *P. fluorescens* CICC23251, 6: *P. fluorescens* CICC23254, 7: *P. aeruginosa* CICC10351, 8: *Pseudomonas aeruginosa* ATCC 27853, 9: *P. aeruginosa* ATCC 15692, 10: *Pseudomonas alcaligenes* CICC23927, 11: *P. alcaligenes* CICC 23069, 12: *P. alcaligenes* CICC25146, 13: *P. simiae* CICC22692, 14: *Pseudomonas stutzeri* CICC10402, 15: *P. stutzeri* CICC23002, 16: *Pseudomonas chlororaphis* CICC21627, 17: *P. chlororaphis* ATCC 13986, 18: *P. chlororaphis* CICC 10216, 19: *P. rhodesiae* CICC21957, 20: *Pseudomonas rhodesiae* CICC 22694, 21: *P. rhodesiae* CICC 22695, 22: *P. putida* ATCC17485, 23: *P. putida* CICC21624, 24: *P. putida* ATCC 12633, 25: *P. putida* ATCC 23483, 26: *Bacillus cereus* CICC20450, 27: *B. cereus* CICC10468, 28: *B. cereus* ATCC 14579, 29: *B. cereus* ATCC 11778, 30: *Bacillus licheniformis* ATCC21424, 31: *B. licheniformis* CICC10037, 32: *B. licheniformis* CICC22068, 33: *B. licheniformis* ATCC 14580, 34: *Staphylococcus aureus* ATCC25923, 35: *S. aureus* ATCC6538, 36: *S. aureus* CICC 10145, 37: *S. aureus* CICC 10201, 38: *Listeria monocytogenes* ATCC19111, 39: *L. monocytogenes* ATCC 51772, 40: *L. monocytogenes* ATCC 19115, 41: *Cronobacter sakazakii* ATCC29544, 42: *C. sakazakii* CICC 24112, 43: *C. sakazakii* CICC 24125, 44: *S. typhimurium* CICC21484, 45: *S. typhimurium* CICC 21913, 46: *S. typhimurium* CICC 21483, 47: Blank control.

To determine the specificity of the RealAmp assay, 2 *P. lurida* strains and 44 related strains were analyzed. Based on the primers designed for the *gyrB* gene, the fluorescence intensity of 2 *P. lurida* strains showed an S-type amplification curve with the extension of amplification time, and the results were positive. In contrast, the fluorescence intensity (delta Rn) of non-*P. lurida* strains and the blank control were fixed at approximately zero; no amplification reaction occurred, and the results were negative (Figure 2A).

The dissociation temperature of the amplified products of *P. lurida* was similar to that of the *gyrB* gene, which was approximately 85.0°C. The dissociation temperatures of the positive amplification products of *P. lurida* CICC22026 and CICC22027 were 85.18°C and 85.49°C, respectively. There was no dissociation temperature for non-*P. lurida* and blank control samples (Figure 2B). This evidenced that the entire amplification reaction was carried out under normal conditions, and no non-specific amplification reactions had occurred.

**Figure 3:**
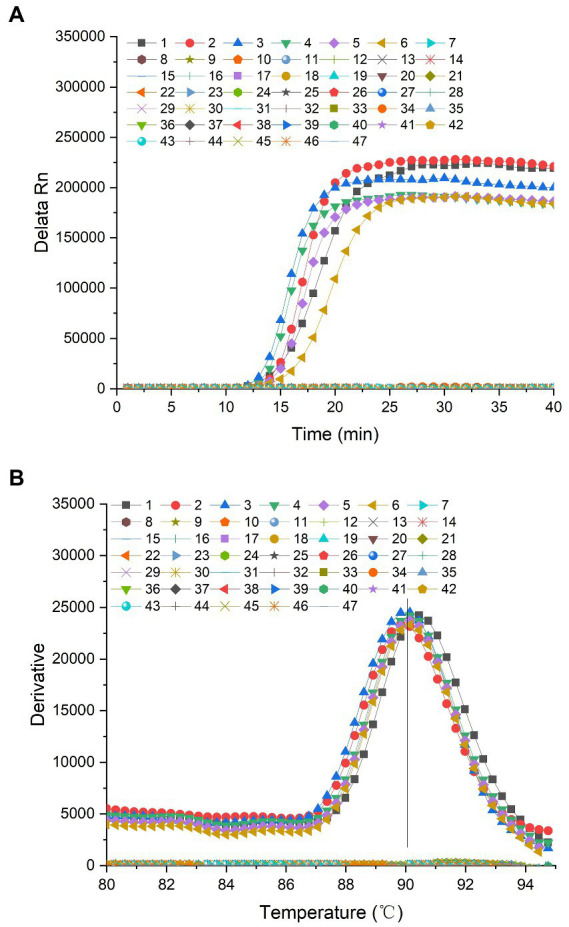
**(A)** Amplification curve of the specificity of the *aprX* gene of *Pseudomonas lurida*. 1: *P. lurida* CICC22026, 2: *P. lurida* CICC22027, 3: *Pseudomonas fluorescens* ATCC13525, 4: *P. fluorescens* CICC23250, 5: *P. fluorescens* CICC23251, 6: *P. fluorescens* CICC23254, 7: *Pseudomonas aeruginosa* CICC10351, 8: *P. aeruginosa* ATCC 27853, 9: *P. aeruginosa* ATCC 15692, 10: *Pseudomonas alcaligenes* CICC23927, 11: *P. alcaligenes* CICC 23069, 12: *P. alcaligenes* CICC25146, 13: *Pseudomonas simiae* CICC22692, 14: *Pseudomonas stutzeri* CICC10402, 15: *P. stutzeri* CICC23002, 16: *Pseudomonas chlororaphis* CICC21627, 17: *P. chlororaphis* ATCC 13986, 18: *P. chlororaphis* CICC 10216, 19: *Pseudomonas rhodesiae* CICC21957, 20: *P. rhodesiae* CICC 22694, 21: *P. rhodesiae* CICC 22695, 22: *P. putida* ATCC17485, 23: *P. putida* CICC21624, 24: *P. putida* ATCC 12633, 25: *P. putida* ATCC 23483, 26: *Bacillus cereus* CICC20450, 27: *B. cereus* CICC10468, 28: *B. cereus* ATCC 14579, 29: *B. cereus* ATCC 11778, 30: *B. licheniformis* ATCC21424, 31: *B. licheniformis* CICC10037, 32: *B. licheniformis* CICC22068, 33: *B. licheniformis* ATCC 14580, 34: *Staphylococcus aureus* ATCC25923, 35: *S. aureus* ATCC6538, 36: *S. aureus* CICC 10145, 37: *S. aureus* CICC 10201, 38: *Listeria monocytogenes* ATCC19111, 39: *L. monocytogenes* ATCC 51772, 40: *L. monocytogenes* ATCC 19115, 41: *Cronobacter sakazakii* ATCC29544, 42: *C. sakazakii* CICC 24112, 43: *C. sakazakii* CICC 24125, 44: *Salmonella typhimurium* CICC21484, 45: *S. typhimurium* CICC 21913, 46: *S. typhimurium* CICC 21483, 47: Blank control. **(B)** Dissociation curve of the specificity of the *aprX* gene of *Pseudomonas lurida*. 1: *P. lurida* CICC22026, 2: *P. lurida* CICC22027, 3: *Pseudomonas fluorescens* ATCC13525, 4: *P. fluorescens* CICC23250, 5: *P. fluorescens* CICC23251, 6: *P. fluorescens* CICC23254, 7: *Pseudomonas aeruginosa* CICC10351, 8: *P. aeruginosa* ATCC 27853, 9: *P. aeruginosa* ATCC 15692, 10: *Pseudomonas alcaligenes* CICC23927, 11: *P. alcaligenes* CICC 23069, 12: *P. alcaligenes* CICC25146, 13: *P. simiae* CICC22692, 14: *Pseudomonas stutzeri* CICC10402, 15: *P. stutzeri* CICC23002, 16: *P. chlororaphis* CICC21627, 17: *P. chlororaphis* ATCC 13986, 18: *P. chlororaphis* CICC 10216, 19: *Pseudomonas rhodesiae* CICC21957, 20: *P. rhodesiae* CICC 22694, 21: *P. rhodesiae* CICC 22695, 22: *P. putida* ATCC17485, 23: *P. putida* CICC21624, 24: *P. putida* ATCC 12633, 25: *P. putida* ATCC 23483, 26: *Bacillus cereus* CICC20450, 27: *B. cereus* CICC10468, 28: *B. cereus* ATCC 14579, 29: *B. cereus* ATCC 11778, 30: *B. licheniformis* ATCC21424, 31: *B. licheniformis* CICC10037, 32: *B. licheniformis* CICC22068, 33: *B. licheniformis* ATCC 14580, 34: *Staphylococcus aureus* ATCC25923, 35: *S. aureus* ATCC6538, 36: *S. aureus* CICC 10145, 37: *S. aureus* CICC 10201, 38: *Listeria monocytogenes* ATCC19111, 39: *L. monocytogenes* ATCC 51772, 40: *L. monocytogenes* ATCC 19115, 41: *Cronobacter sakazakii* ATCC29544, 42: *C. sakazakii* CICC 24112, 43: *C. sakazakii* CICC 24125, 44: *S. typhimurium* CICC21484, 45: *S. typhimurium* CICC 21913, 46: *S. typhimurium* CICC 21483, 47: Blank control.

Only 2 *P. lurida* strains and 4 *P. fluorescens* strains were amplified based on the primers designed for the *aprX* gene. In contrast, the fluorescence intensity of non-*P. lurida* strains and the blank control were fixed at approximately zero, and no amplification reaction occurred (Figure 3A). These results indicate that the primers designed for the *aprX* gene cannot distinguish *P. lurida* and *P. fluorescens* from related strains during RealAmp detection. Therefore, the species-specific detection of *P. lurida* for the *gyrB* gene was the key in RealAmp detection. In addition, the detection of *P. fluorescens* by targeting the *gyrB* gene should be carried out simultaneously to determine whether the thermostable alkaline proteases were produced by *P. lurida* or *P. fluorescens*.

The dissociation temperature of the amplified products of *P. lurida* and *P. fluorescens* was similar to that of the *aprX g*ene, which was approximately 90.0°C. The dissociation temperatures of the positive amplification products of *P. lurida* (CICC22026 and CICC22027) and *P. fluorescens* (ATCC13525, CICC23246, CICC23248, CICC23250, CICC23251, and CICC23254) were 90.36°C, 90.18°C, 90.07°C, 90.36°C, 90.18°C, and 90.49°C, respectively. There were no dissociation temperatures for non-*P. lurida* and blank control samples (Figure 3B). This evidenced that the whole amplification reaction was carried out under normal conditions, and no non-specific amplification reaction had occurred.

### 3.3. Limit of detection in pure culture and skimmed milk samples

**Figure 4:**
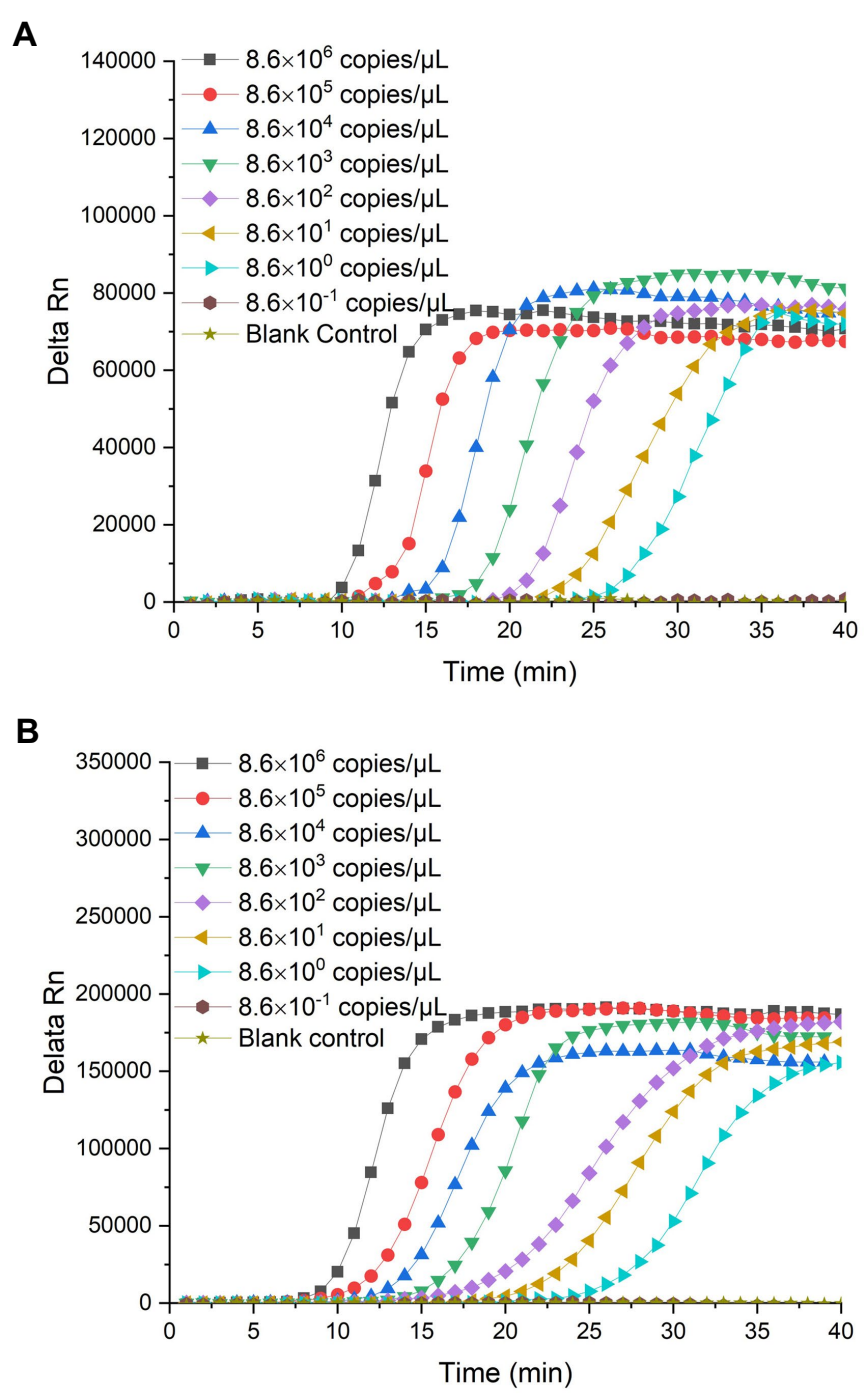
**(A)** Detection limit of *Pseudomonas lurida* in pure culture using RealAmp based on the *gyrB* gene. **(B)** Detection limit of *Pseudomonas lurida* in pure culture using RealAmp based on the *aprX* gene.

**Figure 5:**
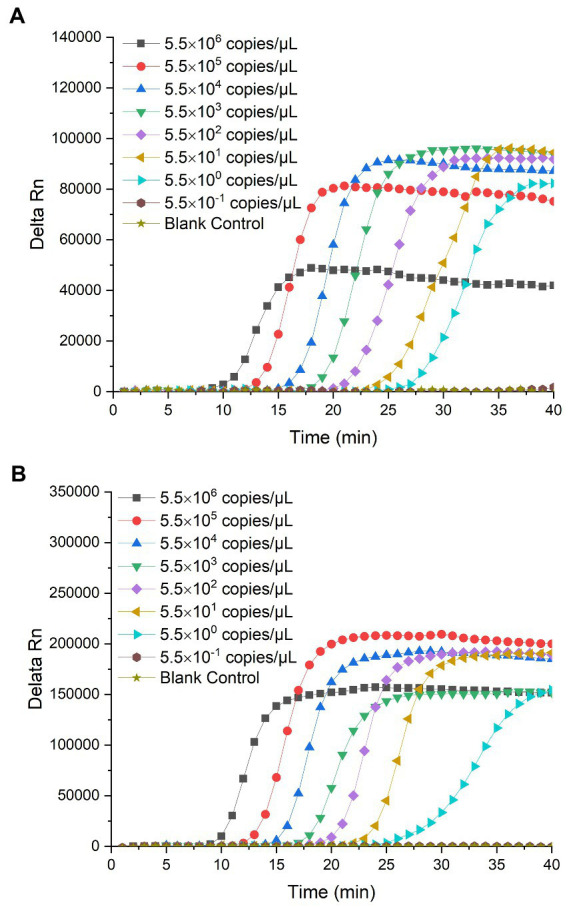
**(A)** Detection limit of *Pseudomonas lurida* in 10% skimmed milk using RealAmp based on the *gyrB* gene. **(B)** Detection limit of *Pseudomonas lurida* in 10% skimmed milk using RealAmp based on the *aprX* gene.

The limit of detection of the RealAmp assay was determined by detecting *P. lurida* in pure culture and artificially contaminated 10% skimmed milk samples at a continuous 10-fold dilution. The lower the value of detection limit, the higher the sensitivity of the RealAmp method. The RealAmp limit of detection of *P. lurida* based on the g*yrB* and *aprX* genes was 5.6 × 10^2^ CFU/ml (57.5 fg/μL, i.e., 8.6 copies/μL) in pure culture (Figures 4A,B), whereas that in artificially contaminated 10% skimmed milk was 2.4 × 10^2^ CFU/ml (37.0 fg/μL, i.e., 5.5 copies/μL; Figures 5A,B). Furthermore, the detection limit of the RealAmp assay in skimmed milk for detection of *P. lurida* and its thermostable alkaline proteases was almost the same or lower than that in pure culture, which indicated that the food matrix had no effect on the detection ability of RealAmp.

The CV values of the Ct for the 10 tests of *P. lurida* pure culture medium were less than 2%, ranging between 0.60 and 1.68%, and 0.77 and 1.96%, respectively (Table 1). Further, the CV values of the peak emergence time of *P. lurida* in skimmed milk were also less than 2%, ranging between 0.92 and 1.86%, and 0.77 and 1.90%, respectively (Table 2). These results indicate that the RealAmp method has good repeatability.

**Table 1:** Reproducibility results of the limit of detection of *Pseudomonas lurida* in pure culture solutions using RealAmp based on the *gyrB* and *aprX* genes.

Order no.	DNA copy number (copies/μL)	Duplicate numbers detected	Detection based on *gyrB* gene *via* RealAmp			Detection based on *aprX* gene *via* RealAmp		
			Mean	SD	CV(%)	Mean	SD	CV(%)
1	8.6 × 10^6^	10	9.96	0.17	1.68	9.67	0.19	1.96
2	8.6 × 10^5^	10	12.06	0.18	1.46	12.31	0.24	1.95
3	8.6 × 10^4^	10	14.79	0.20	1.34	15.01	0.18	1.20
4	8.6 × 10^3^	10	17.78	0.22	1.22	17.54	0.20	1.14
5	8.6 × 10^2^	10	20.66	0.12	0.60	20.25	0.17	0.84
6	8.6 × 10^1^	10	23.16	0.26	1.12	23.47	0.18	0.77
7	8.6 × 10^0^	10	26.50	0.31	1.19	26.43	0.27	1.02

Mean: average Ct value of 10 detections; SD: average standard deviation of 10 Ct values; CV: coefficient of variation of 10 Ct values.

**Table 2:** Reproducibility results of the limit of detection of *Pseudomonas lurida* in skimmed milk solutions using RealAmp based on the *gyrB* and *aprX* genes.

Order no.	DNA copy number (copies/μL)	Duplicate numbers detected	Detection based on *gyrB* gene *via* RealAmp			Detection based on *aprX* gene *via* RealAmp		
			Mean	SD	CV (%)	Mean	SD	CV (%)
1	5.5 × 10^6^	10	10.89	0.16	1.50	9.43	0.14	1.48
2	5.5 × 10^5^	10	13.24	0.25	1.86	12.11	0.23	1.90
3	5.5 × 10^4^	10	16.42	0.19	1.18	15.02	0.18	1.20
4	5.5 × 10^3^	10	18.73	0.22	1.15	17.98	0.17	0.95
5	5.5 × 10^2^	10	21.41	0.20	0.92	20.21	0.22	1.09
6	5.5 × 10^1^	10	24.81	0.28	1.12	23.17	0.21	0.91
7	5.5 × 10^0^	10	27.41	0.32	1.16	26.12	0.20	0.77

Mean: average Ct value of 10 detections; SD: average standard deviation of 10 Ct values; CV: coefficient of variation of 10 Ct values.

### 3.4. *Pseudomonas lurida* detection in real raw milk samples

The average detection rate of the *gyrB* gene of *P. lurida* in raw milk throughout the year was 45%, and that of *aprX* was 26%, indicating that raw milk is susceptible to contamination by *P. lurida*. The detection rate of *P. lurida*-positive in the four quarters of the year was also analyzed (Table 3); the detection rates in the first, second, third, and fourth quarters were 56, 44, 56, and 25%, respectively. The detection rate of *P. lurida* in the first and third quarters was significantly higher than that in the fourth quarter (*p* < 0.05); however, there was no significant difference among the other quarters (*p* > 0.05). Moreover, there was no significant difference in the detection ratio of *aprX* among the different quarters (*p* > 0.05). The detection rate of the thermostable alkaline proteases of *P. lurida* in all quarters was lower than that of *P. lurida* itself, and the highest ability of *P. lurida* to produce thermostable alkaline proteases was observed in the second quarter (81%). The highest detection rate of *P. lurida* and thermostable alkaline proteases appeared in different seasons.

**Table 3:** RealAmp detection results of *Pseudomonas lurida* in raw milk samples collected throughout the year.

Quarter	The number of positive detections		Positive detection rate (%; *n* = 36)		Protease-producing ability of *P. lurida* (%)
	*gyrB gene*	*aprX gene*	*P. lurida*-positive	*aprX*-positive	*aprX*-positive/*P. lurida*-positive
First (1–3 months)	20	8	56	22	40
Second (4–6 months)	16	13	44	36	81
Third (7–9 months)	20	12	56	33	60
Fourth (10–12 months)	9	4	25	11	44

*n* = 36: 36 samples were tested each quarter.

In addition, the detection rate of *P. lurida*-positive in raw milk obtained from three pastures in the Hebei province was analyzed (Table 4). The detection ratios of pastures 1, 2, and 3 were 46, 52, and 38%, respectively; however, there was no significant difference in the detection ratio among raw milk samples from the different pastures. In contrast, the detection ratio of *aprX* gene in pasture 2 was significantly higher than that in pastures 1 and 3 (*p* < 0.05). Moreover, the detection rate of the thermostable alkaline proteases of *P. lurida* in the 3 pastures was lower than that of *P. lurida* itself, and the highest ability of *P. lurida* to produce thermostable alkaline proteases was observed in pasture 2 (88%). The highest detection rate of *P. lurida* and thermostable alkaline proteases both appeared in the same pasture.

**Table 4:** RealAmp detection results of *Pseudomonas lurida* in raw milk samples collected from different pastures.

Pasture	The number of positive detection		Positive detection rate (%; *n* = 48)		Protease-producing ability of *P. lurida* (%)
	*gyrB gene*	*aprX gene*	*P. lurida*-positive	*aprX*-positive	*aprX*-positive/*P. lurida*-positive
1	22	9	46	19	41
2	25	22	52	46	88
3	18	6	38	13	33

*n* = 48: 48 samples were collected from each pasture.

## 4. Discussion

At present, there are few studies on *P. lurida*, and its potential harm to food is easy to ignore, particularly the damage it causes to raw milk stored at low temperatures. In addition, the development of rapid detection methods for this bacterium is still limited. In the present study, we developed a fluorescent RealAmp method with high sensitivity and specificity to detect *P. lurida* and its thermostable alkaline proteases. This method enables the early detection and control of contamination to ensure the quality of milk and dairy products and reduce economic losses of enterprises.

In the current study, the highest *P. lurida*-positive detection ratio was observed in the first and third (56%), followed by the second (44%) and fourth (25%) quarters of the year. These data show that the growth of *P. lurida* was suitable for nearly 3/4 seasons in the Hebei province. Like *P. fluorescens*, *P. lurida* has become the main psychrophilic bacterium affecting the quality of raw milk in Hebei province of China. This observation is consistent with the growth characteristics of *P. lurida* as a psychrophilic bacterium, growing at a wide temperature range of 4–30°C and having an optimal growth temperature of 28°C (Vithanage et al., 2016). The environment in the first, second, and third quarters may be more suitable for *P. lurida* growth, which explains the higher *P. lurida* detection rates in these quarters compared to those in the fourth quarter. In addition, the ability of *P. lurida* to produce proteases detected in raw milk samples in the second (81%) and third (60%) quarters was significantly higher than that of the first (40%) and fourth (44%) quarters, showing a strong correlation with quarters. Additionally, the range of temperatures at which proteases were produced was narrower than the range of the growth temperature of *P. lurida*. The optimal temperature for protease production was also lower than that of *P. lurida* growth, and considerably low temperatures were not conducive to the production of *P. lurida* thermostable alkaline proteases. These results are consistent with previous research (Nucera et al., 2016; Yuan et al., 2018). In a previous study, *P. lurida* was isolated from raw milk and cultured at 7°C and 28°C, and proteases were produced at both temperatures; however, the ability of *P. lurida* to produce proteases at 7°C was lower than that at 28°C (Yuan et al., 2018). Similarly, another study reported protease production by *P. lurida* isolates from milk samples at both 5°C and 25°C (Nucera et al., 2016). These results and our study suggest that reducing the storage temperature of raw milk to reduce the ability of *P. lurida* to produce proteases may be an effective way to control protease production in raw milk. However, the types and heat resistance of the proteases produced by the isolated *P. lurida* were not further studied in the present study and, therefore, warrant future investigation.

The biodiversity of psychrophilic bacteria in raw milk in Shanghai, China was studied by Xu et al. (2017) through the use of 16S rRNA high-throughput sequencing. The results showed that *Pseudomonas* was the main group of psychrophilic bacteria, accounting for 55% of the total number of psychrophilic bacteria, of which 60% showed protease activity. *Pseudomonas* spp. appears in raw milk every season, which indicates that it is a bacterium, and that its thermostable alkaline proteases may play an important role in raw milk spoilage (Jonghe et al., 2011; Li et al., 2019). A previous study analyzed the microbial diversity of year-round raw milk from three pastures in the Hebei province by using high-throughput sequencing, detected the bacteria responsible for contaminating raw milk at different times, and investigated the correlation between these bacteria and raw milk quality parameters (Yuan et al., 2022). The results showed that psychrophilic bacteria in all three pastures were a serious threat to the quality of raw milk. Psychrotolerant *Pseudomonas* species are the main cause of proteolytic spoilage of ultra-high temperature milk products because of the production of the thermostable alkaline proteases, which is encoded by the first gene of the aprX-lipA2 operon (Maier et al., 2020). The phylogenomic position and aprX-lipA2 gene organization specify the proteolytic potential of *Pseudomonas* isolates. In addition, however, an interplay of several environmental factors and intrinsic traits influences the production and activity of thermostable alkaline proteases (Zhang et al., 2019, 2020).

In the present study, raw milk samples from three farms (48 samples from each farm) were analyzed. The detection rate of *P. lurida*-positive was the highest in pasture 2 (52%), which may be attributed to the pasture environment. *Pseudomonas lurida* is present in the surrounding environment, such as the water, soil, and cushion materials (Masiello et al., 2014); therefore, poor hygiene in pastures may lead to *P. lurida* proliferation. In addition, raw milk is temporarily stored in a refrigeration tank; therefore, the cold-phillic bacterium *P. lurida* can continue to reproduce during the refrigeration period of raw milk, causing contamination. In the present study, the ability of *P. lurida* to produce thermostable alkaline proteases was also the highest in pasture 2 (88%). This ability is related to the characteristics of the *P. lurida* strain; some *P. lurida* strains have the *aprX* gene, which can produce proteases, while other strains lack this gene and, therefore, do not produce proteases (Caldera et al., 2016; Xu et al., 2017; Guo, 2020). In a recognition study, 116 strands of *Pseudomonas* spp. were isolated from raw milk in Heilongjiang, Inner Mongolia, Gansu, Henan, Anhui, Jiangsu, Huanan, and Chongqing in China. The findings revealed that 60.3% of *Pseudomonas* spp. carried the *aprX* gene, and the activity of proteolytic enzymes was different among the regions (Du, 2022). In our study, the protease-producing ability of the *P. lurida* strain isolated from the samples from pasture 2 was higher than that of the samples from pastures 1 and 3. These results suggest that protective measures, such as cleanliness, need to be improved to control the risk of contamination.

In a previous study, positive numbers of thermostable alkaline proteases were detected using RealAmp with primers designed for the *apr*X gene, which are the total numbers of thermostable alkaline proteases-producing samples of *P. lurida* and *P. fluorescens* (Hu et al., 2022). However, RealAmp detection with primers designed for the *gyrB* gene of *P. lurida* and *P. florescens* exhibited no cross-reaction and had a specificity of 100% (Hu et al., 2022). Therefore, the positive samples with thermostable alkaline proteases corresponded to their positive-producing bacteria, *P. lurida* or *P. florescens*. Three sets of primers were amplified using RealAmp in three reaction tubes, which could simultaneously identify *P. lurida* and *P. florescens* as well as their thermostable alkaline proteases. From each tube, 1 μL DNA was extracted and amplified using three sets of primers systems. However, the test results showed that the positive tubes of *P. lurida* and *P. florescens* completely corresponded to the positive samples of thermostable alkaline proteases produced by their respective bacteria, without cross-amplification. Therefore, we believe that if the DNA extract is sufficiently uniform, RealAmp would detect the DNA of the same sample in three reaction tubes (one set of primers/tube), which is equivalent to detecting the DNA of the same sample in one reaction tube (three sets of primers/tube).

## 5. Conclusion

The present study established a rapid RealAmp detection method for *P. lurida* and its thermostable alkaline proteases. The detection limits in 10% skimmed milk within 40 min were 5.5 copies/μL. This method rapidly detected and analyzed the contamination with *P. lurida* and its thermostable alkaline proteases in raw milk samples collected from three pastures throughout the year. This rapid method provides a scientific basis for enterprises to identify and control contamination sources in a timely manner to ensure the quality of milk and dairy products.

## Data availability statement

The data presented in this study are deposited in the NCBI GenBank repository, accession number MT941328.1.

## Author contributions

All the authors contributed to the conception and design of the study. LH and YX conceived the study. SZ and LH performed the experiments. SZ and DZ analyzed the data. YZ and SW developed the methodology. SZ and LH wrote the manuscript. All authors contributed to the article and approved the submitted version.

## Funding

This work was supported by the National Key R&D Program of China (grant 2018YFC1604305), the Program of Hebei Provincial Department of Science and Technology (19222805D), the Natural Science Foundation of Hebei Province (C2022106010), the Key Research and Development Projects of Shijiazhuang (211170212A), and the Doctoral Research Startup Fund Project of Shijiazhuang University (grant number 20BS007).

## Conflict of interest

Author(s) YX, DZ, YZ, and SW were employed by Junlebao Dairy Group Co., Ltd.

The remaining authors declare that the research was conducted in the absence of any commercial or financial relationships that could be construed as a potential conflict of interest.

## Publisher’s note

All claims expressed in this article are solely those of the authors and do not necessarily represent those of their affiliated organizations, or those of the publisher, the editors and the reviewers. Any product that may be evaluated in this article, or claim that may be made by its manufacturer, is not guaranteed or endorsed by the publisher.
